# Supplementary material for: An Evidence and Consensus-Based Definition of Second Victim: A Strategic Topic in Healthcare Quality, Patient Safety, Person-Centeredness and Human Resource Management
Source: Int J Environ Res Public Health. 2022 Dec 15;19(24):16869. doi: 10.3390/ijerph192416869 (PMC9779047; doi:10.3390/ijerph192416869)
Supplement: Supplementary file 1 [file ijerph-19-16869-s001.zip › ijerph-2063986-supplementary.pdf]

Table S1: Details of the included studies
* NM: not mentioned

Study	Country	Study design	Respondents	Sample size	Setting	Timing of study*
Abusalem et al. 2013 [36]	USA	Observational study	Home nurses	192	Outside hospital	NM
Ajoudani et al. 2020 [37]	Iran	Observational study	Nurses	298	In-hospital (multicenter)	NM
Ajri-Khameslou et al. 2017 [38]	Iran	Qualitative study	Emergency nurses	18	In-hospital	2014-2015
Bari et al 2016 [39]	India	Observational study	Pediatric medicine residents	130	In-hospital	NM
Biggs et al. 2020 [40]	Great Britain and Ireland	Observational study	Surgeons	82	In-hospital (multicenter)	NM
Brunelli et al 2018 [41]	South America	Validation study	Nurses	169	In-hospital	NM
Burlison et al. 2016 [42]	USA	Observational study	Nurses	155	In-hospital	NM
Burlison et al. 2017 [43]	USA	Validation study	Staff members	305	In-hospital	NM
Cebeci et al. 2015 [44]	Turkey	Observational study	Nursing students	324	School of nursing	2009
Chan et al. 2018 [45]c	Singapore	Qualitative study	Nurses	8		NM
Chen et al. 2019 [46]	China	Validation study	Nurses	1442		NM
Choi et al. 2020 [47]	Korea	Observational study	Nurses	492	In-hospital	2019
Christoffersen et al. 2020 [48]	Norway	Qualitative study	Midwives	33	In-hospital (multicenter)	2014
Chung et al. 2018 [49]	Worldwide	Qualitative study	Residents			2016-2017
Deringer et al. 2014 [50]	USA	Qualitative study	Psychiatric residents	64	In-hospital	2012-2013
Dukhanin et al. 2018 [51]	USA	Experimental study	Nurses, physicians, mangers and others	+/-200	In-hospital	2011-2014
Edrees et al. 2017 [52]	USA	Qualitative study	Patient safety representatives	43	In-hospital (multicenter)	2013-2014
Edrees et al. 2017 [53]	USA	Qualitative study	Patient safety representatives	43	In-hospital (multicenter)	2013-2014
Edrees et al. 2011 [54]	USA	Observational study	Healthcare workers	350	In-hospital	NM
Edrees et al. 2016 [55]	USA	Observational study	Risk managers	641	In-hospital (multicenter)	2013
Edrees et al. 2016 [56]	USA	Experimental study	Staff members	+/-500	In-hospital	2010-2012
El Hechi et al. 2020 [57]	Canada	Experimental study	Surgeons and surgical trainees	47 interventions	In-hospital	NM
Finney et al. 2020 [58]	USA	Observational study	Nurses	115	In-hospital	2019
Graham et al. 2019 [59]	USA	Experimental study	Physicians and staf	44	In-hospital	NM
Gupta et al. 2019 [60]	USA	Observational study	Physician mothers	5782	in-hospital	2016

Habibzadeh et al. 2020 [61]	Iran	Observational study	Nurses	298	In-hospital (multicenter)	NM
Han et al. 2017 [62]	USA	Observational study	Surgeons	126	In-hospital (multicenter)	NM
Harrison et al. 2014 [63]	UK	Observational study	Physicians	1755		2013
Harrison et al. 2015 [64]	UK and USA	Observational study	Physicians and nurses	265	In-hospital (multicenter)	NM
Harrison et al. 2018 [7]	Australia and New Zealand	Observational study	Anaesthists	247	In-hospital	2016-2017
Harrison et al. 2019 [65]	Vietnam	Observational study	Doctors and nurses	497	In-hospital	NM
Huang et al. 2020 [66]	China	Observational and qualitative study	Nursing students	1417		2019-2020
Joesten et al. 2015 [67]	USA	Observational study	Healthcare professionals	120	In-hospital	2011
Kable et al. 2018 [68]	Australia	Qualitative study	Nurses	10	In-hospital	NM
Kim et al. 2020 [69]	Korea	Validation study	Nurses	305	In-hospital	2016
Kobe et al. 2019 [70]	Canada	Observational and qualitative study	Radiation therapists	178		NM
Koehn et al. 2016 [71]	USA	Qualitative study	Nurses	13	In-hospital	NM
Krzan et al. 2015 [72]	USA	Experimental study	Pharmacy staff	121	In-hospital	2013
Lane et al. 2018 [73]	USA	Experimental study	Residents, fellows, faculty members, NPs/Pas and CRNAs	165	In-hospital	2014-2017
Luu et al. 2012 [74]	Canada	Qualitative study	Surgeons	20		NM
McCarthy et al. 2016 [75]	Ireland	Experimental study	Junior doctors	208		2011-2013
McLennan et al. 2015 [76]	Switzerland	Observational study	Anaesthesiologist	281	In-hospital (multicenter)	2012-2013
Merandi et al. 2017 [77]	USA	Qualitative study	Healthcare workers	232	In-hospital	2012-2016
Merandi et al. 2018 [5]	USA	Experimental study	Nurses, APN, therapist, unit clerk, physician, manager, patient care assistant, unit technician, dietician, pharmacist, service coordinator, other	250	In-hospital	2016
Mira et al. 2015 [6]	Spain	Observational study	Doctors, nurses other	1087	In -and out-hospital	NM
Mira et al. 2015 [78]	Spain	Observational study	Managers and patient safety coordinations	406	In -and out-hospital	2014
Mohamadi-Bolbanabad et al. 2019 [79]	Iran	Observational study	Physicians, nurses and midwives	338	In-hospital	2017
Mohsenpour et al. 2018 [80]	Iran	Qualitative study	Nurses	8	In-hospital (multicenter)	NM

Mok et al. 2020 [81]	Singapore	Observational study	Nurses	1163	In-hospital	2018
Panella et al. 2016 [19]	Italy	Observational study	Physicians	1313	In-hospital	2014
Pijl Zieber et al. 2015 [82]	Canada	Qualitative study	Nursing students	16		NM
Pinto et al. 2014 [83]	UK	Observational study	General and vascular surgeons	47	In-hospital	NM
Pratt et al. 2015 [84]	USA	Qualitative study	Panel of stakeholders	14		NM
Pyo et al. 2020 [85]	Korea	Observational study	Physicians	895		NM
Quillivan et al. 2016 [86]	Columbia	Observational study	Nurses	155	In-hospital	2013
Reiser et al. 2020 [87]	Switzerland	Observational study	Hospitals' quality managers and chief executive officers	116	In-hospital	2018
Rinaldi et al. 2016 [18]	Italy	Qualitative study	Nurses, physicians and other healthcare workers	33	In-hospital	2012-2013
Scheepstra et al. 2020 [88]	The Netherlands	Observational study	Gynaecologists, orthopaedic surgeons, pediatricians	1374	In-hospital	NM
Schrøder et al. 2019 [89]s	Denmark	Observational study	Midwives and obstetricians	2098		2012
Stangierski et al. 2012 [90]	Poland	Observational study	Doctors	100		NM
Stone 2019 [91]	USA	Qualitative study	Nurses			NM
Stone 2020 [92]	USA	Qualitative study	Nurses	12	In-hospital	NM
Strametz et al. 2021 [93]	Germany	Validation study	Nurses, physicians, medical assistants, physician assistants, paramedics, medical therapists and remedial therapist	306	In- and out-hospital	2020
Strametz et al. 2021 [94]	Germany	Observational study	Physicians	555	In-hospital	2019
Stukalin et al. 2019 [95]	Canada	Observational study	Physicians	51		NM
Swartwout et al. 2017 [96]	USA	Validation study	Nurses	497		NM
Tan et al. 2020 [97]	China	Validation study	Nurses	731		NM
Treiber et al. 2018 [98]	USA	Observational study	Nursing graduates	168		NM
Tumelty 2018 [12]	Ireland	Qualitative study	Medical training and legal professionals	18		2015
Ullström et al. 2014 [99]	Sweden	Qualitative study	Healthcare professionals	21	In-hospital	2011
Van Gerven et al. 2016 [17]	Belgium	Observational study	Physicians, nurses and midwives	913	In-hospital	NM
Van Gerven et al. 2016 [16]	Belgium	Qualitative study	Physicians, nurses and midwives	31		2012-2013

Van Gerven et al. 2016 [4]	Belgium	Observational study	Physicians and nurses	5788	In-hospital	NM
Vanhaecht et al. 2019 [22]	The Netherlands	Observational study	Doctors and nurses	4369	In-hospital	NM
Vinson et al. 2014 [100]	USA	Observational study	Anesthesia residence		In-hospital	NM
Wahlberg et al. 2020 [101]	Sweden	Qualitative study	Midwives and obstetricians	14	In-hospital	NM
White et al. 2015 [102]	USA	Observational study	US members of ASHRM	635		2013
Winning et al. 2018 [103]	USA	Observational study	healthcare providers in neonatal intensive care unit	463	In-hospital	2015
Winning et al. 2020 [104]	USA	Validation study	Healthcare providers in neonatal intensive care units	514	In-hospital	NM
Yung et al. 2016 [105]	Taiwan	Observational study	Nurses	306	In-hospital	NM
Zeeman et al. 2020 [27]	The Netherlands	Observational study	Nurses and doctors	2635	In-hospital (multicenter)	2016-2017
Zhang et al. 2020 [106]	China	Validation study	Nurses	625	In-hospital (multicenter)	NM
Zhang et al. 2019 [107]	China	Observational study	Nurses	267	In-hospital (multicenter)	2017

Table S2: Different concepts used in definitions and/or descriptions of SV

Who was involved? (Specific defined in definition/description)
Healthcare provider/healthcare staff/healthcare worker/healthcare professional (n:66)<br><br>Physician (n:23)/Surgeons (n:1)/Anesthesists (specialists and trainee) (n:1)<br><br>Nurse (n:10)<br><br>Clinician (n:8)<br><br>Involved healthcare professional/healthcare provider (n:4)<br><br>Surgeons (n:3)<br><br>Healthcare team member (n:3)<br><br>Medical staff/medical professional (n:3)<br><br>Caregivers and staff (n:2)<br><br>Directly or indirectly contributed (n:2)<br><br>Dedicated medical staff (n:1)<br><br>Medical practitioners (n:1)<br><br>Pharmacists (n:1)<br><br>Other providers (n:1)<br><br>Student (n:1)<br><br>Residents (n:1)
What had happened?
Medical error (n:30)/Unanticipated medical error (n:8)/ Unexpected medical error (n:1)<br><br>Adverse event (n:26)/ Serious adverse event (n:2)/adverse event that cause lesions or other types of harm or suffering in patients (n:1)/adverse event that injure patients in their care (n:1)/unexpected adverse event (n:1)/ Unpredictable adverse event (n:1)/ Unforeseen patient adverse event (n:1)/unforeseen adverse event and or harm suffered by the patient (n:1)/ Unanticipated patient-related adverse event (n:1)/ Medical adverse event (n:1)/ Intraoperative adverse event (n:1)<br><br>Patient related injury (n:11)/ Patient related injury even not directly involved in patient outcome or as near miss even in cases where the event never reaches the patient (n:1)<br><br>Unanticipated patient event (n:4)<br><br>Unexpected adverse patient safety incident (n:3)/ Patient safety incident (defined as event or circumstances that could have resulted, or did result, in unnecessary harm to a patient (n:3)/patient safety incident (adverse event occur due to treatment involved and medical error) (n:4)<br><br>Adverse patient-related event (n:3)

Near-miss adverse event (n:3)<br><br>Unanticipated clinical events or outcome (n:2)/Adverse clinical event (n:1)<br><br>Non-error patient safety event (n:1)/ Patient safety event (n:2)<br><br>Mistake (n:2)<br><br>Unanticipated stressfull situation (n:1)/ Stressfull situation (n:1)/stressfull event (n:1)<br><br>Adverse incident (n:1)<br><br>Unanticipated adverse patient event (n:1)<br><br>Serious near-miss patient safety event (n:1)<br><br>Safety event that results in harm to a patient (n:1)<br><br>Same incident that harm the patient (n:1)<br><br>Patient harm (n:1)/Patient associated harm (n:1)<br><br>Other patient related injury (n:1)<br><br>Harming a patient (n:1)<br><br>Event (n:4)<br><br>Unexpected negative patient outcome (n:2)/unanticipated patient outcome (n:1)<br><br>Unanticipated patient harm (n:1)<br><br>Error (n:6)/error resulting in patient injury or death (n:1)/ Nursing errors (n:1)/ Patient care error (n:1)<br><br>Can occur even when no error has occurred but the patient has unfavourable outcome (n:1)<br><br>Adverse outcome (n:1)/patient adverse outcome (n:1)/ Psychiatric adverse outcome (n:1)<br><br>Death or injury because of medical error (n:1)
Result of what had happened?
Trauma (n:49)<br><br>Emotional impact (n:45)<br><br>Victimized (n:16)/victimized of stress (n:1)<br><br>Psychological impact (n:16)<br><br>Negative impact (n:13)<br><br>Affected (n:12)<br><br>Personally responsible (n:11)<br><br>Long-term effect (n:11)

Personal impact (n:9)
Failed patient (n:8)
Suffer (n:7)
Professional impact (n:6)
Physical impact (n:6)
Disruption (n:6)
Responsible (n:3)
Damage (n:1)
